# The gendered effects of the COVID-19 pandemic on adolescent literacy and schooling outcomes in India

**DOI:** 10.1038/s41539-023-00193-8

**Published:** 2023-09-22

**Authors:** Arindam Nandi, Nicole Haberland, Meredith Kozak, Thoại D. Ngô

**Affiliations:** 1 The Population Council, Dag Hammarskjold Plaza, New York, NY 10017 USA; 2 One Health Trust, Washington, DC USA

**Keywords:** Education, Economics

## Abstract

The COVID-19 pandemic disrupted education delivery around the world, with school closures affecting over 1.6 billion students worldwide. In India, schools were closed for over 18 months, affecting 248 million students. This study estimates the effect of the pandemic on adolescent literacy and schooling outcomes in India. We used data from the National Family Health Survey. (NFHS-5) which covered 636,699 households across all districts of India from June 2019 to April 2021. We considered 15–17 year old adolescents who were surveyed after March 2020 as the post-COVID group while those surveyed earlier were included in the pre-COVID group. We used propensity score matching and inverse propensity score weighted regression methods to account for differences in socioeconomic characteristics between the two groups. Rates of literacy (ability to read a complete sentence) were 1.5–1.6% lower among post-COVID girls as compared with similar pre-COVID girls. Among post-COVID girls in the lowest wealth quintile, rates of literacy were 3.1–3.8% lower than similar pre-COVID girls. There was no loss in literacy among post-COVID girls in the highest wealth quintile. COVID-induced loss in literacy among girls was twice in rural areas as compared to urban areas, and substantially higher among socioeconomically disadvantaged caste groups as compared with privileged caste groups. Post-COVID girls also had 0.08–0.1 lower years of schooling completed than similar pre-COVID girls but there was no difference in out-of-school rates. In a smaller subsample of 15–17 year old boys, the post-COVID group had 2% lower out-of-school rates and there was no difference in literacy or years of schooling completed as compared with matched pre-COVID boys. While markers of vulnerability such as residence, caste, and poverty further amplified the risk of learning. loss for girls, they did not have the same effect on boys.

## Introduction

India is home to an estimated of 20% of the world’s adolescent population—equivalent to 253 million 10–19 year old boys and girls^1^. Even before the COVID-19 pandemic, foundational literacy and numeracy skills among Indian adolescents were less than universal. In 2018, only 73% and 44% of Indian children in the eighth grade (age 14) could read second-grade level text and solve a simple numerical division problem, respectively^2^. There were also substantial gender gaps in arithmetic skills and out-of-school rates in favor of boys in the 14–16 year age group^2^.

The pandemic has disrupted education systems globally, with lockdowns and school closures resulting in approximately half a year’s worth of learning loss—with substantial variation across countries—among students^3^. Like many governments around the world, the government of India responded to the early stages of the pandemic with non-pharmaceutical interventions (NPIs) such as lockdowns, travel restrictions, and directives for social distancing. India entered a 21-day national lockdown on 24th March 2020. All business and administrative establishments other than essential services such as grocery stores, hospitals, and banks were closed, all gatherings were prohibited or restricted, and public transit was suspended. Schools and colleges were closed, and remote learning curricula were introduced by national and state education agencies.

Although many NPI restrictions in India were gradually lifted starting in June 2020, educational institutions continued to be closed. The states of Delhi, Rajasthan, Tamil Nadu, Uttar Pradesh, and Madhya Pradesh were the first to reopen schools (with COVID mitigating measures) in early September 2021. Schools reopened on a rolling basis across other states over the next few months, with the national government issuing guidelines for school reopening to all states in February 2022^4^. By this time, children in India had lost over 18 months of schooling—the second longest COVID-related school closures in the world after Uganda—with varying levels of access to remote learning^5^.

School closures severely disrupted teaching and learning. The Annual Status of Education Report (ASER)—a leading rural education survey conducted annually in India—examined schooling and learning among 59,000 children in 9000 schools across the country in September 2020^6,7^. The study found that although a digital curriculum was introduced during the pandemic, 38% of 6–14-year-old children enrolled in schools did not have access to a smartphone at home. Among children enrolled in private schools, 29% and 18% reported receiving lessons from school through recorded video and online classes respectively, while among government school children, these rates were only 18% and 8%. Only 50% of teachers reported receiving any form of training for online teaching, and during the week preceding the survey, 33% of teachers reported delivering no instructions to students at all. Availability of learning tools varied widely across states, e.g., >80% of enrolled students in Gujarat, Kerala, and Punjab received some learning material (other than textbooks) from school during the week preceding the survey, as compared with fewer than 25% of students in Bihar, Rajasthan, Uttar Pradesh, and West Bengal^6,7^.

The ASER 2023 report included findings from their nationwide schooling and learning survey from 2022 which was the first full-scale survey of 6–14-year-old Indian children since 2018 (the 2019 survey covered early childhood education while surveys during the pandemic were smaller and conducted by phone)^8^. The authors found that despite the pandemic, school enrollment rate in the 6–14 year age group increased from 97.2% in 2018 to 98.4% in 2022, and the out-of-school rate among 15–16 year girls reduced from 13.5% to 7.9%. However, there were substantial reductions in standardized test scores across all ages during this time. Among students in grade 8, the proportion of those who could read basic text (grade 2 level) reduced from 73% to 69.6%, and in younger grades, the reductions in reading capability were even higher. Another regional study from Tamil Nadu estimated COVID-related learning losses of 0.7 and 0.34 standard deviations for mathematics and language respectively among 5–7 year old students^9^.

A key limitation of the ASER is that the study does not collect data on—and adjust for –children’s background socioeconomic characteristics. Intergenerational mobility in human capital development—e.g., improved schooling and learning outcomes among children of richer or more educated parents—has been well documented in India^10–14^. The adverse effects of the pandemic on learning outcomes might be stronger among socioeconomically disadvantaged children, but national estimates have yet to be available. Furthermore, considering that boys in India are more likely to be enrolled in better quality private schools while girls are more likely to be enrolled in government schools, the learning loss could be higher among girls^2^. The Tamil Nadu study accounted for background characteristics of children and also found no gender or socioeconomic status gaps in learning loss but these findings may not be generalizable to the rest of the country^9^.

Learning loss due to the pandemic is estimated to reduce future economic productivity by $98.84 billion by 2030 in India^15^. Educational policymakers require data and evidence on which segments of children are the most affected by the pandemic, so that targeted policies can be designed for a resilient, equitable, and inclusive education delivery system. To our knowledge, ours is the first study to address the limitations of the previous literature and examine the effects of the pandemic on learning and schooling outcomes of 15–17-year-old girls and boys in India.

We used data from the National Family Health 2019–2021 (NFHS-5), which collected nationally representative information on ~100,000 girls of age 15–17 years. Data from a smaller but nationally representative sample of 14,000 boys of this age were also available and used. We considered those surveyed prior to the first COVID-19 national lockdown (25 March 2020) in the pre-COVID group, while those surveyed later were included in the post-COVID group. We controlled for differences in background socioeconomic characteristics of the two groups by employing quasi-experimental methods—propensity score matching (PSM) and inverse propensity score weighted (IPW) regression—that are widely used to estimate causal relationships in observational data, conditional upon certain underlying assumptions^16–19^. We matched each post-COVID adolescent with one or more observationally similar pre-COVID adolescent and evaluated the effect of the pandemic on reading ability, schooling enrollment and attainment, and the reasons for being out of school. We present estimates separately by location, caste, religion, and wealth quintiles, subject to statistical power limitations for subsample analysis for boys.

## Results

### Characteristics of the study sample

Table 1 presents the summary statistics of the pre-COVID (surveyed before 25 March 2020, the first day of the national lockdown) and post-COVID (surveyed on or after 25 March 2022) samples of adolescent girls and boys. There were 32,936 and 66,994 girls of age 15–17 years in the post-COVID and pre-COVID groups, respectively. At the time of the survey, there were 27,018 out-of-school girls (of whom 8917 were in the post-COVID group). Girls in the post-COVID group were 2.6% less likely to be able to read as compared with girls in the pre-COVID group. There were no statistically significant differences in school enrollment or years of schooling completed between the two groups. Among girls who were out of school, the post-COVID group reported the cost of schooling as the reason at higher rates than the pre-COVID group, and reported marriage or employment as the reason at lower rates. The post-COVID group was 3.7% more likely to be from the highest wealth quintile (wealth quintile 5) as compared with the pre-COVID group. They were also less likely to be from rural areas and minority religions. There were some other differences (e.g., caste groups and relationship to household head indicators) which were statistically significant but small in magnitude.

**Table 1:** Summary statistics of the sample—15–17 year old girls and boys in India.

	15–17 year old girls			15–17 year old boys		
	Pre-COVID	Post-COVID	Difference (Post–Pre)	Pre-COVID	Post-COVID	Difference (Post–Pre)
Reading ability (can read?)	0.819 (0.385)	0.793 (0.405)	−0.026**	0.824 (0.381)	0.821 (0.383)	−0.003
Currently out of school	0.27 (0.444)	0.271 (0.444)	0.001	0.239 (0.427)	0.223 (0.416)	−0.017*
Out of school for marriage	0.165 (0.371)	0.085 (0.279)	−0.08**	–	–	–
Out of school for employment	0.171 (0.377)	0.148 (0.355)	−0.024**	0.177 (0.382)	0.171 (0.377)	−0.006
Out of school due to cost	0.16 (0.366)	0.231 (0.421)	0.071**	0.136 (0.343)	0.186 (0.389)	0.05**
Age in years	16.503 (1.137)	16.551 (1.124)	0.049**	16.479 (1.127)	16.553 (1.105)	0.074**
Years of schooling completed	8.97 (2.693)	8.936 (2.762)	−0.034	8.874 (2.737)	8.932 (2.637)	0.059
HAZ	−1.901 (0.004)	−1.884 (0.006)	−0.017*	−0.442 (0.014)	−0.259 (0.021)	−0.383**
Relationship to household head:						
Self	0.002 (0.049)	0.002 (0.043)	−0.001	0.005 (0.067)	0.003 (0.052)	−0.002
Spouse	0.012 (0.107)	0.006 (0.078)	−0.005**	0 (0)	0 (0)	0
Daughter	0.777 (0.416)	0.788 (0.409)	0.011**	0.829 (0.377)	0.816 (0.387)	−0.012
Daughter-in-law	0.047 (0.212)	0.031 (0.172)	−0.017**	0.001 (0.031)	0.001 (0.033)	0
Grandchild	0.107 (0.309)	0.117 (0.322)	0.01**	0.114 (0.318)	0.132 (0.338)	0.018**
Age of household head	48.85 (11.373)	48.83 (11.187)	−0.02	48.762 (11.355)	49.099 (11.258)	0.337
Whether household head is female	0.164 (0.37)	0.15 (0.357)	−0.014**	0.169 (0.375)	0.163 (0.369)	−0.006
Household head’s years of schooling	5.599 (5.414)	5.655 (5.736)	0.057	5.741 (5.848)	5.796 (5.287)	0.055
Household characteristics:						
Household size	5.727 (2.278)	5.936 (2.375)	0.208**	5.375 (2.196)	5.507 (2.229)	0.132**
Rural	0.784 (0.411)	0.775 (0.417)	−0.009**	0.767 (0.423)	0.76 (0.427)	−0.008
Scheduled Caste (SC)	0.2 (0.4)	0.231 (0.421)	0.031**	0.193 (0.395)	0.215 (0.411)	0.022**
Scheduled Tribe (ST)	0.182 (0.386)	0.199 (0.4)	0.017**	0.184 (0.388)	0.197 (0.397)	0.012
Other Backward Classes (OBC)	0.371 (0.483)	0.401 (0.49)	0.03**	0.38 (0.486)	0.409 (0.492)	0.029**
Muslim	0.165 (0.371)	0.104 (0.305)	−0.061**	0.149 (0.356)	0.088 (0.284)	−0.061**
Christian	0.083 (0.276)	0.032 (0.175)	−0.052**	0.086 (0.281)	0.03 (0.171)	−0.056**
Sikh	0.016 (0.124)	0.024 (0.153)	0.008**	0.019 (0.135)	0.029 (0.169)	0.011**
Wealth quintile 1 (poorest)	0.234 (0.423)	0.272 (0.445)	0.038**	0.214 (0.41)	0.259 (0.438)	0.045**
Wealth quintile 2	0.257 (0.437)	0.231 (0.422)	0.026**	0.254 (0.435)	0.221 (0.415)	−0.034**
Wealth quintile 3	0.219 (0.413)	0.186 (0.389)	−0.033**	0.227 (0.419)	0.18 (0.384)	−0.047**
Wealth quintile 4	0.175 (0.38)	0.159 (0.366)	−0.016**	0.177 (0.382)	0.17 (0.376)	−0.007
Wealth quintile 5 (richest)	0.115 (0.319)	0.152 (0.359)	0.037**	0.127 (0.333)	0.17 (0.376)	0.043**
Sample size	66,994	32,936		9217	4497	

Data are from National Family Health Survey of India, 2019–2021 (NFHS-5). Standard deviations are in the parenthesis. SC, ST, and OBC are government of India designated socioeconomically disadvantaged caste groups. HAZ denotes height-for-age z score. *p < 0.05, **p < 0.01.

There were 4497 boys of age 15–17 years in the post-COVID group and 9217 boys in the pre-COVID groups. Among them, 1001 and 2204 boys across the two groups were out of school respectively. There were no statistically significant differences in reading ability or years of schooling completed between the two groups, while post-COVID boys were 1.7% less likely to be out of school as compared with the pre-COVID group. Out-of-school boys in the post-COVID group had higher rates of reporting the cost of schooling as the reason for school leaving than the pre-COVID group. The post-COVID group was more likely to be from richer and Hindu households. There were smaller but significant differences in a few additional indicators.

### Estimates of the effect of the pandemic

PSM (with nearest neighbor and three nearest neighbors) and IPW regression estimates of the effect of the pandemic on adolescent girls’ and boys’ reading ability are presented in Table 2. Among girls, the ability to read a sentence in the post-COVID group ranged from 1.5%–1.6% lower as compared with the matched pre-COVID group. There was substantial variation in the estimated effect of the pandemic on girl’s reading ability by socioeconomic subgroups. The effect among girls in rural areas (1.8% reduction in reading ability) was about twice that of girls in urban areas, and among caste groups, socioeconomically disadvantaged other backward classes (OBC) had the highest reduction in reading ability after the pandemic (1.5%–1.6% reduction). Across wealth quintiles, the estimated effect was largest among girls in the poorest wealth quintile (quintile 1) (3.1%–3.8% reduction in reading ability), and it ranged from 0.9%–2.1% in wealth quintiles 2, 3, and 4. No statistically significant effect was seen in the richest wealth quintile (quintile 5). There was no significant difference in the reading ability of boys between the matched pre- and post-COVID groups, except in the upper caste and fourth wealth quintile subsamples where the post-COVID boys had lower reading ability.

**Table 2:** Propensity score matching based estimates of the effect of the COVID-19 pandemic on adolescent girls’ and boys’ (15–17 years old) reading ability in India.

	15–17 year old girls				15–17 year old boys			
	Nearest neighbor matching	Three nearest neighbors matching	Inverse propensity score weighted regression	Sample size	Nearest neighbor matching	Three nearest neighbors matching	Inverse propensity score weighted regression	Sample size
Full sample	−0.016 (0.003)**	−0.015 (0.003)**	−0.015 (0.002)**	94,868	−0.013 (0.009)	−0.008 (0.007)	−0.005 (0.006)	12,773
Rural	−0.011 (0.004)**	−0.018 (0.003)**	−0.017 (0.002)**	74,517	0.008 (0.011)	0 (0.008)	−0.002 (0.007)	9842
Urban	−0.009 (0.006)	−0.007 (0.005)	−0.008 (0.004)*	20,351	−0.004 (0.018)	−0.016 (0.013)	−0.018 (0.011)	2931
SC or ST	−0.012 (0.005)*	−0.012 (0.004)**	−0.015 (0.004)**	38,054	−0.012 (0.015)	−0.017 (0.011)	−0.008 (0.01)	5011
OBC	−0.015 (0.005)**	−0.013 (0.004)**	−0.017 (0.003)**	36,036	−0.003 (0.014)	−0.006 (0.011)	−0.004 (0.009)	4955
Upper Caste	−0.005 (0.007)	−0.005 (0.005)	−0.008 (0.004)	16,194	−0.033 (0.016)*	−0.02 (0.013)	−0.012 (0.012)	2228
Hindu	−0.015 (0.004)**	−0.018 (0.003)**	−0.016 (0.002)**	71,108	−0.001 (0.009)	−0.003 (0.008)	−0.007 (0.006)	9750
Other Religions	−0.025 (0.008)**	−0.024 (0.006)**	−0.016 (0.005)**	23,760	0.01 (0.023)	−0.007 (0.018)	−0.004 (0.015)	3023
Wealth Quintile 1	−0.031 (0.008)**	−0.038 (0.007)**	−0.034 (0.005)**	23,422	−0.024 (0.021)	−0.03 (0.017)	−0.028 (0.015)	2938
Wealth Quintile 2	−0.015 (0.007)*	−0.01 (0.005)	−0.016 (0.004)**	23,724	0.001 (0.019)	0.008 (0.015)	0.009 (0.012)	3139
Wealth Quintile 3	−0.021 (0.006)**	−0.016 (0.005)**	−0.009 (0.004)*	19,831	0.015 (0.017)	0.007 (0.013)	0.007 (0.012)	2698
Wealth Quintile 4	−0.021 (0.006)**	−0.018 (0.005)**	−0.009 (0.004)*	16,160	−0.037 (0.014)**	−0.028 (0.012)*	−0.006 (0.012)	2218
Wealth Quintile 5	0 (0.006)	0.003 (0.005)	0.002 (0.004)	11,731	0.006 (0.019)	0.007 (0.014)	−0.005 (0.011)	1780

Data are from the National Family Health Survey of India, 2019–2021 (NFHS-5). Standard errors are in the parenthesis. SC, ST, and OBC are government of India designated socioeconomically disadvantaged caste groups. *p < 0.05, **p < 0.01.

Estimates of the effect of the pandemic on out-of-school rates, years of schooling completed, and reasons for not being enrolled in school are presented in Table 3. Girls in the post-COVID group had 0.08–0.1 fewer schooling years completed as compared with girls in the matched pre-COVID group. While there were no statistically significant differences in PSM models, the IPW regression estimates showed that post-COVID boys had 0.1 fewer schooling years completed as compared to the matched pre-COVID group. There was no difference in out-of-school rates between pre- and post-COVID girls, while post-COVID boys were 2% less likely to be out of school as compared with matched pre-COVID boys. Among out-of-school post-COVID girls, 3.5%–3.9% and 3.2%–3.6% fewer girls reported marriage and employment as the reasons for being out of school respectively, as compared with the matched pre-COVID group. Post-COVID girls also reported the cost of schooling as the reason for being out of school at 6% higher rates than the matched pre-COVID group. Similarly, out-of-school post-COVID boys reported the cost of schooling as the reason at 5–6% higher rates as compared to boys in the matched pre-COVID group.

**Table 3:** Propensity score matching based estimates of the effect of the COVID-19 pandemic on adolescent outcomes in India.

	15–17 years old girls	Sample size	15–17 years old boys	Sample size
Nearest neighbor PSM:				
Years of schooling completed	−0.104 (0.025)**	95,117	−0.116 (0.066)	12,799
Currently out of school	−0.002 (0.004)	95,117	−0.016 (0.01)	12,799
Out of school for marriage	−0.035 (0.005)**	25,794	–	
Out of school for employment	−0.036 (0.007)**	25,794	0.002 (0.022)	2964
Out of school due to cost	0.056 (0.007)**	25,794	0.068 (0.019)**	2964
Three nearest neighbor PSM:				
Years of schooling completed	−0.088 (0.02)**	95,117	−0.095 (0.054)	12,799
Currently out of school	−0.006 (0.003)	95,117	−0.024 (0.009)**	12,799
Out of school for marriage	−0.039 (0.004)**	25,794	–	
Out of school for employment	−0.033 (0.006)**	25,794	0 (0.018)	2964
Out of school due to cost	0.059 (0.006)**	25,794	0.055 (0.017)**	2964
IPW regression:				
Years of schooling completed	−0.084 (0.017)**	95,117	−0.097 (0.046)*	12,799
Currently out of school	−0.005 (0.003)	95,117	−0.022 (0.008)**	12,799
Out of school for marriage	−0.037 (0.003)**	25,794	–	
Out of school for employment	−0.032 (0.005)**	25,794	−0.004 (0.016)	2964
Out of school due to cost	0.058 (0.005)**	25,794	0.052 (0.015)**	2964

Data are from National Family Health Survey of India, 2019–2021 (NFHS-5). Standard errors are in the parenthesis. PSM denotes propensity score matching and IPW denotes inverse propensity score weighted regression. *p < 0.05, **p < 0.01.

**Fig. 1:**
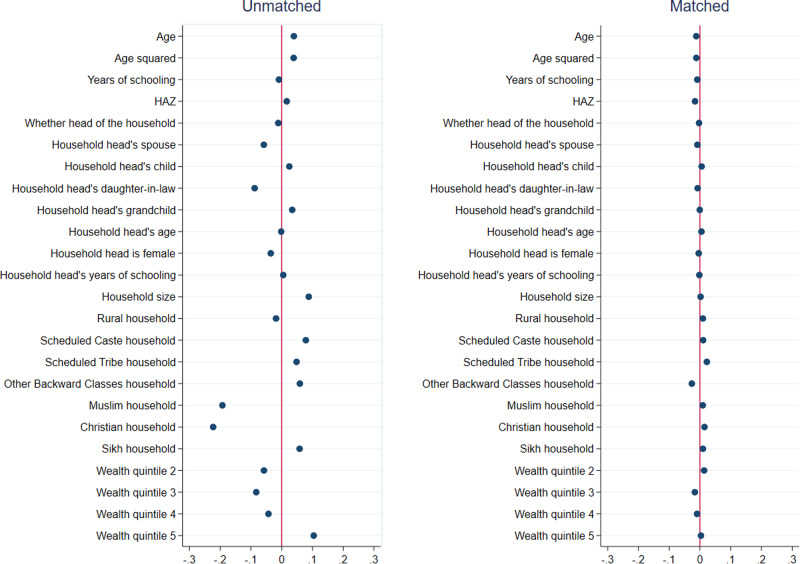
Covariate balance—nearest neighbor propensity score matching, reading ability of 15–17 year old girls. Data are from National Family Health Survey of India, 2019–2021 (NFHS-5). Standardized percentage bias in covariates (difference between post- and pre-COVID groups) are show separately in unmatched data and after nearest neighbor matching. HAZ height-for-age z score.

**Fig. 2:**
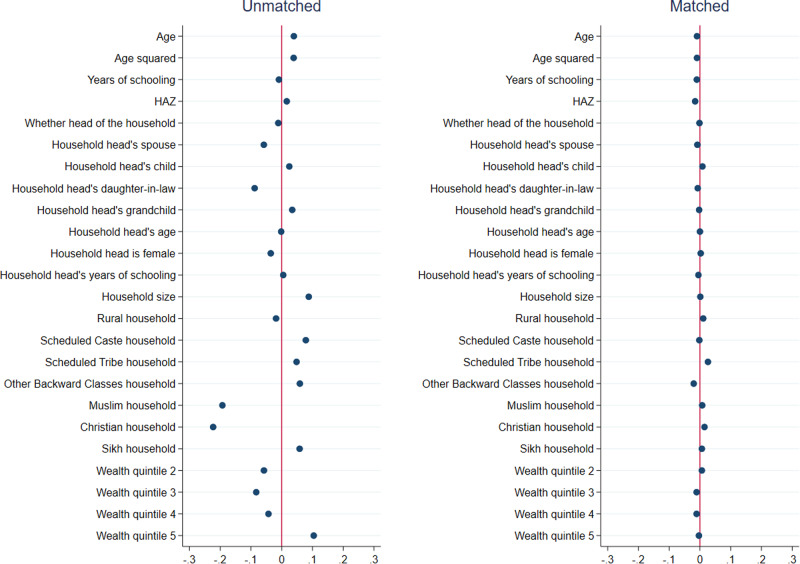
Covariate balance—three nearest neighbors propensity score matching, reading ability of 15–17 year old girls. Data are from National Family Health Survey of India, 2019–2021 (NFHS-5). Standardized percentage bias in covariates (difference between post- and pre-COVID groups) are show separately in unmatched data and after three nearest neighbors matching. HAZ height-for-age z score.

**Fig. 3:**
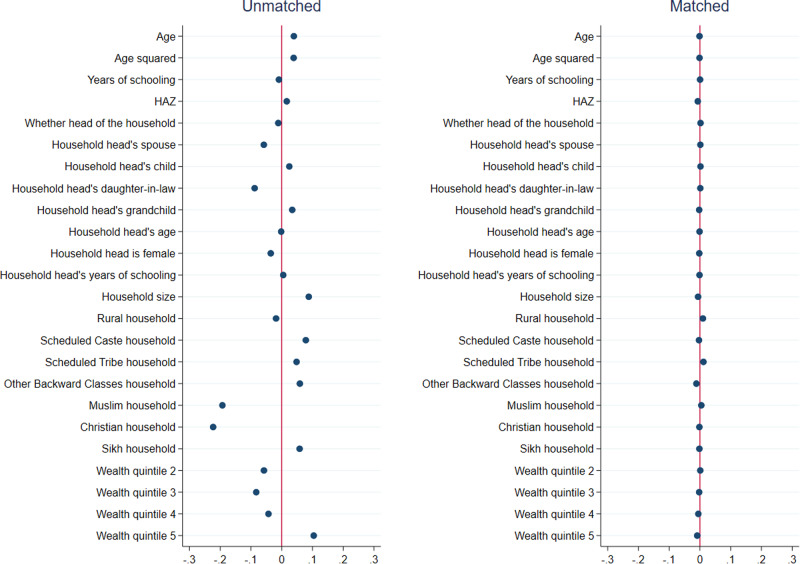
Covariate balance—inverse propensity score weighted regression, reading ability of 15–17 year old girls. Data are from National Family Health Survey of India, 2019–2021 (NFHS-5). Standardized percentage bias in covariates (difference between post- and pre-COVID groups) are show separately in unmatched data and after inverse propensity score weighted regression. HAZ height-for-age z score.

**Fig. 4:**
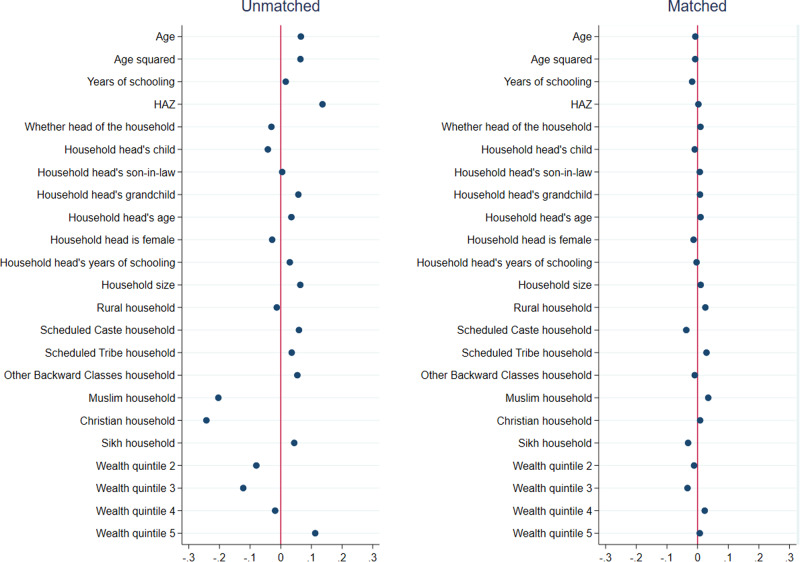
Covariate balance—nearest neighbor propensity score matching, reading ability of 15–17 year old boys. Data are from National Family Health Survey of India, 2019–2021 (NFHS-5). Standardized percentage bias in covariates (difference between post- and pre-COVID groups) are show separately in unmatched data and after inverse propensity score weighted regression. HAZ height-for-age *z* score.

**Fig. 5:**
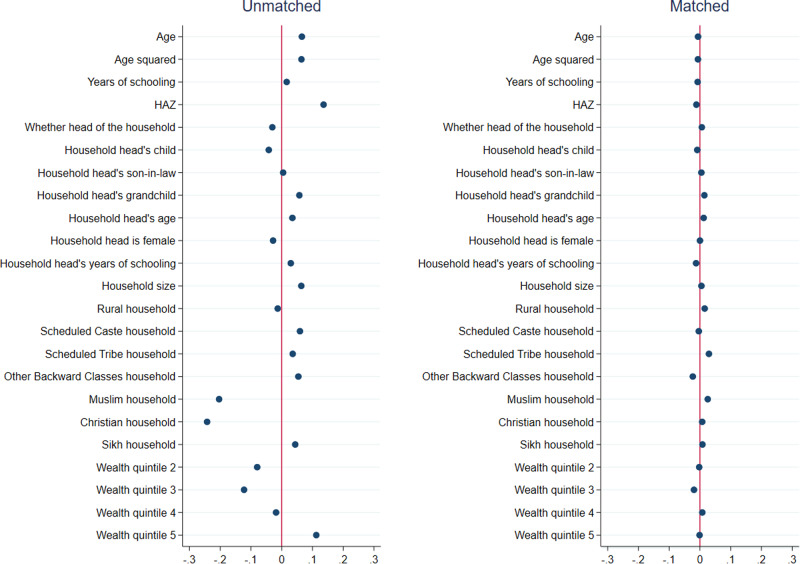
Covariate balance—three nearest neighbors propensity score matching, reading ability of 15–17 year old boys. Data are from National Family Health Survey of India, 2019–2021 (NFHS-5). Standardized percentage bias in covariates (difference between post- and pre-COVID groups) are show separately in unmatched data and after inverse propensity score weighted regression. HAZ height-for-age z score.

**Fig. 6:**
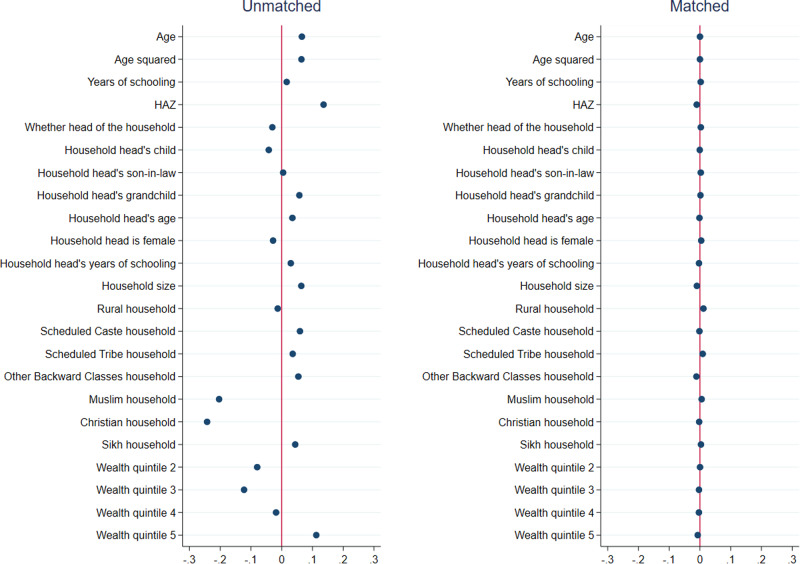
Covariate balance—inverse propensity score weighted regression, reading ability of 15–17 year old boys. Data are from National Family Health Survey of India, 2019–2021 (NFHS-5). Standardized percentage bias in covariates (difference between post- and pre-COVID groups) are show separately in unmatched data and after inverse propensity score weighted regression. HAZ height-for-age z score.

Results from matching quality (covariate balance) tests for girls are presented in Figs. 1–3, and those for boys are presented in Figs. 4–6. In each figure, the left panel shows the standardized percentage bias—which measures the difference between post- and pre-COVID groups—for each covariate before PSM. The standardized percentage bias after PSM was employed is shown in the right panel. To preserve space, we only present results from the analysis of reading ability. PSM for other outcome indicators yielded similar results (not shown). All three matching methods reduced biases from 0–3% in the unmatched data to close to 0 in the matched sample, with IPW being the most effective in reducing bias. This implies that our methodological approach was valid.

## Discussion

We used nationally representative data from NFHS-5 to estimate the effect of the pandemic on reading ability, years of schooling attained, and reasons for school leaving on adolescent girls and boys ages 15–17 in India. Girls who were surveyed after the start of the pandemic had lower reading ability and schooling attainment as compared with girls who were observationally similar but were surveyed before the pandemic. The reduction in reading ability from the pre- to the post-pandemic period was the highest among girls in the lowest wealth quintile. Boys did not experience any reduction in literacy or in schooling years attained due to the pandemic, and they were less likely to be out-of-school after the pandemic. Strikingly, all markers of marginalization we examined—residence, caste, and class—increased vulnerability to pandemic-related learning loss among girls, but not among boys.

Our findings align with results of the ASER 2022 survey although there are some differences attributable to the study period and methodology. In our unmatched data, there was a reduction in reading ability (can read a sentence) from 81.9% to 79.3% from 2019 to 2021, although based on two different samples of 15–17-year-old girls, and no reduction for boys. In comparison, ASER noted a reduction in reading ability (can read grade 2 level text, one paragraph) among girls in grade 8 (age 14 years) from 73.6% in 2018 to 71.3% in 2022, while among grade 8 boys, it reduced from 72.1% to 67.6%^8^. After accounting for socioeconomic factors using propensity score matching analysis, we found 1.5–1.6% reduction in the reading ability of girls but no reduction among boys during 2019–2021. We also found no change in out-of-school rates for girls and a 2% reduction among boys, as compared with ASER which found reductions during 2018–2022 for both sexes.

There were no significant differences in out-of-school rates of girls pre- and post-pandemic, and literacy rates in the pre-pandemic baseline were high (82% were able to read). Therefore, pandemic-related school closures were likely the main reason for the reductions in reading ability. Schools were closed from the first national lockdown through the entire period during which the NFHS-5 data (second phase) were collected. One might expect that foundational learning, once acquired, is an asset that cannot be taken away, and that by ages 15–17 an adolescent either can or cannot read. Our findings suggest, however, that even during mid-adolescence, reading skills may be tenuous. In addition, poor and marginalized girls in India are at a disadvantage, with access to worse quality schools and lower levels of academic achievement and foundational learning than richer girls^6,7^. In our study, only 64% of girls in the poorest wealth quintile before the pandemic were able to read, and they experienced the largest decline in reading ability due to school closures. Similar learning loss has been documented for girls who left school in Malawi, even without the precipitating impact of a pandemic^20^.

The differences in the estimated effect of the pandemic on the outcomes of girls and boys may be driven partly by variations in sample sizes. While the survey was nationally representative for both girls and boys, the sample size of 13,714 boys might have had inadequate statistical power to detect very small changes in outcome indicators. This is especially true for subgroup analysis where the sample size is reduced further. The estimated effect of the pandemic on boys’ outcomes were negative and similar in magnitude as seen among girls (Tables 2 and 3), and if the sample size were larger, the estimates could have been statistically significant.

The higher COVID-induced literacy and schooling attainment loss among girls compared to boys could also be explained in part by systemic gender discrimination. There is a strong preference for boys over girls in India, which is manifested through harmful gender norms and practices such as sex selection (including abortion of female fetuses) and lower human capital investment for girls and women (i.e., lower spending for health and education for girls as compared with boys)^21–24^. Girls in India are more likely to be enrolled in government schools while boys are enrolled in higher quality private schools, which contributes to persistent male-biased gender gaps in educational achievement^2,25–30^. Gender bias was also evident in access to digital learning tools and time use even before the pandemic. Analysis of the National Sample Survey of 2017–2018 (75th round special survey on education) data show that after controlling for socioeconomic indicators, 10–19-year-old boys were 3–7% more likely to have access to the internet than girls of that age^31^. The 2017 national ASER survey in rural India estimated that 14–18-year-old boys had 10 and 27 percentage point higher access to a mobile phone and the internet respectively than 14–18-year-old girls^32^. Similarly, analysis of National Family Health Survey 2005–2006 (NFHS-3) data show that 5–14–year old girls in India were substantially more likely to be engaged in household chores than boys of the same age^33^.

The pandemic effectively eliminated one of the ways that girls in India are permitted mobility and access to social and community environments outside of the household—namely, by going to school. Two 2020 studies covering eight states in India found an 11–12 percentage point advantage favoring boys in access to digital learning devices^34–36^. An estimated 32% of adolescent girls aged 10–19 years did not get adequate meals during the pandemic as compared with 27% of boys of the same age^35^. Unequal gender norms and roles also persisted during the pandemic in terms of mobility and responsibilities: girls were involved in household chores 4–33 percentage points more than boys and only 39% of girls were allowed to go outside of home alone compared to 62% of boys^35^. Beyond India, female disadvantage in access to digital learning tools during the pandemic were also observed in other countries such as Bangladesh^37^.

Beyond learning, our analysis reveals other dimensions of adolescent education and well-being. Even before the pandemic, adolescent girls in India were at a high risk of early marriage and dropping out of school^38^. In 2016, an estimated 27% of Indian women of age 20–24 years were married before the age of 18^39^. Among women in this age group, only 10% of those who were married before age 18 years completed secondary schooling as compared with 33% of those who were married after age 18^38^. The proportion of women who never attended any school was also higher among the married before-18 group (25%) as compared with the married-later group (14%).

In 2020, experts raised alarm for an elevated risk of school dropout and child marriage among adolescent girls around the world. An estimated 130 million girls were already out of school worldwide before the start of the pandemic, and an additional 11–20 million girls—primarily from LMICs—were projected to be at risk of dropping out of school and 7–10 million at risk of child marriage due to the pandemic^40–44^. In India, there have been local reports and registered police cases showing a possible rise in child marriages during the pandemic but national estimates have yet to be available^45,46^. Our findings indicate that during the first year of the pandemic, adolescent girls in India were not likely to be out of school at higher rates than the pre-pandemic period. Those who were out of school were less likely to get married and, instead, were not attending school due to high cost of schooling. A new study using the NFHS-5 data has estimated that age of marriage among women in India increased by 3.6% to 19.5 years during the first year of the pandemic, possibly because of delayed weddings due to economic hardship and restrictions on social gatherings^47^. Analyses using longer-term data are necessary to understand the dynamics of child marriage and schooling cessation among adolescent girls during, and following, the pandemic.

Our findings have important policy implications. A 2022 report by the Asian Development Bank estimated the potential economic cost of COVID-19-induced school closures across Asian countries^15^. The authors projected that learning loss among children and associated reduction in their future economic productivity would amount to an economic loss 3.19% of GDP by 2030 in India. This is a substantial loss, equivalent to almost half of the 6.6% GDP loss that India experienced throughout 2020 across all sectors of the economy due to lockdowns and other NPIs^48^.

To avert the massive human capital and economic cost of the pandemic from learning losses, national and state governments must allocate more resources and improve the quality of education delivery. A 2022 study in 41 districts across five states found that supporting teachers to engage with students more in the classroom may help accelerate educational recovery^49^. Another study of 19,000 students aged 5–7 years in Tamil Nadu found that two-thirds of the COVID-related learning loss was recovered within six months of school reopening^9^. The state government’s education recovery program which included after-school learning sessions covering 3.3 million students was responsible for 28% and 21% of the recovery in language and mathematics test scores, respectively^9^. The program cost US$ 7.6 per child per year, and generated a very cost-effective benefit of 3.4 standard deviation gains in test scores per $100. Considering that one standard deviation increase in test scores is estimated to yield a 4.5% rise in wages in LMICs^50^, the aggregate economic benefits of government programs for learning recovery could be very large.

The government of India has taken a step in the right direction by increasing the annual education sector budget by 11.86% from 2021 to 2022 and by another 8% in 2023^51,52^. India launched an ambitious National Education Policy (NEP) in 2020, with emphasis on providing equitable and inclusive access, and improving the quality of education delivery^53^. The increased funding allocation reflects the goals of the NEP, with an effort to help children recover from learning losses. Our findings highlight the importance of the government’s aim of equitable and inclusive access. In India, universal upper secondary schooling completion among girls is estimated to generate $800 million in aggregate economic benefits by 2030^54^. Without targeted policies for mitigating the gendered impact of the pandemic on learning, such benefits may not materialize.

There are some limitations to our analysis. While our matching methods accounted for a wide range of socioeconomic and demographic characteristics of the adolescents, there may be unobserved differences in the characteristics of the pre- and post-COVID groups. If such differences are correlated with learning and schooling outcomes, they might bias our findings. Second, schooling enrollment and attainment outcomes in our data may suffer from measurement errors. The sample of post-COVID adolescents were surveyed during complete school closures, and some respondents may have reported their pre-COVID schooling levels instead of assuming grade progression. Unfortunately, NFHS-5 did not collect additional data on the details of these measurements. Additionally, data on time use or access to digital learning tools, which could help explain the underlying pathways for our findings, were also not available. Finally, the sample size of boys, while representative, was considerably smaller as compared with girls, possibly contributing to the differential effects of the pandemic for girls and boys in our analysis. To ensure that there was no sample selection bias for boys, we examined the characteristics of our study sample for systematic differences with other large household surveys. Supplementary Table 1 shows that the differences in the background characteristics of 15–17-year-old boys and girls have remained similar from the previous round of nationally representative NFHS surveys (NFHS-4, 2015-2016) to NFHS-5.

Despite these limitations, our study shows substantial learning and schooling attainment losses among adolescent girls in India during the first year of the COVID-19 pandemic. Learning loss is heightened by other forms of marginalization for girls, but is insignificant for boys across socioeconomic strata. Remedial education programs, especially for poor and marginalized students, are necessary for improving learning levels and reducing the longer-term risk of dropping out of school.

## Methods

### National Family Health Survey of India 2019–2021 data

We used data from the National Family Health Survey of India, 2019–2021 (NFHS-5)^55^. NFHS surveys in India are part of the Demographic and Health Surveys which are conducted across low- and middle-income countries (LMICs) at regular intervals^56^. NFHS-5 covered all states and territories of India in two phases. The first phase was conducted in 22 states and territories from June 2019 to January 2020, while the second phase covered the remaining 14 states and territories from January 2020 to April 2021. Data collection during the second phase was suspended from April 2020 due to national and regional COVID-19 lockdowns, and the survey resumed in November 2020. The survey covered 2.8 million individuals from 636,699 households across India, of whom 52% were covered in the second phase. NFHS-5 data are publicly available, and ethical approval for data collection was provided by the International Institute for Population Sciences (IIPS) of India. Because this study used publicly available, anonymized, and secondary data from NFHS-5, no separate ethics clearance was necessary.

Four different questionnaires were administered by NFHS-5. A household questionnaire collected information on socioeconomic indicators including location, caste, religion, and ownership of durable assets by the household, along with individual-level information such as age, sex, marital status, and schooling attainment of each household member. Another questionnaire collected data from 724,115 women aged 15–49 years on topics such as sexual and reproductive health including birth history and family planning, and child care, nutrition, and immunization. A third questionnaire was administered to 101,839 men aged 15–54 years and it collected information on topics such as fertility preference, employment, gender attitudes, and HIV/AIDS knowledge. Finally, biomarkers were collected for indicators such as height, weight, hemoglobin, and blood pressure for all respondents.

Our outcome indicator of interest was the reading ability (literacy) of 15–17 year old adolescent girls and boys. The women’s and men’s questionnaires included a literacy card which contained a sentence written in the respondent’s native language. While this information was collected only for individuals who did not complete primary school in previous NFHS surveys, NFHS-5 collected literacy data for everyone. We created a binary indicator of whether the respondent was able to read the complete sentence (as compared to reading partially or not being able to read at all).

We also examined two self-reported education indicators for adolescents: whether they were currently enrolled in (attending) school and their highest grade of schooling completed. Schools in India remained closed from the first national lockdown through almost the end of 2021, after which they started reopening gradually by region. Self-reported schooling data in the post-COVID sample were collected entirely during a period of school closures. Finally, for girls who were out of school, we examined the following binary indicators of their main reason for not being enrolled: (i) whether got married, (ii) needed employment, or (iii) high cost of schooling. Similarly, for boys who were out of school, we evaluated the following reasons: (i) needed employment, or (ii) high cost of schooling. These responses were non-overlapping. We did not examine marriages among boys due to low prevalence rates.

We included those who were surveyed after 25 March 2020 (start date of the first national COVID lockdown) in the post-COVID group while those surveyed earlier were included in the pre-COVID group.

### Analysis

We used PSM methods to estimate the effect of the pandemic on the reading ability of adolescent girls. PSM is a widely used quasi-experimental technique for program evaluation using observational data^16–19^. In our data, the post-COVID and pre-COVID groups may systematically differ in their background characteristics. For example, the post-COVID group is primarily from northern states of India which are more populous and less economically developed compared to southern states. Schooling attainment or reading abilities may be correlated with socioeconomic status, and ordinary least squares estimates of the effect of the pandemic on outcome indicators could therefore be biased (larger in magnitude than any potential true negative effect). PSM can help mitigate such biases by statistically matching each post-COVID observation to a pre-COVID observation of similar characteristics, thus creating homogenous comparison groups.

The first stage of PSM was a logit model, regressing post-COVID status (binary indicator of whether an individual was surveyed by NFHS-5 after 25 March 2020) on a set of individual characteristics which included age in years, squared age, years of schooling completed, and indicators of relationship to household head (whether self, spouse, child, child-in-law, or grandchild). Height-for-age z score was included as a covariate of the regression as it could capture past nutrition, living condition, and other factors that may be correlated with educational outcomes^57,58^. Also included in the regression were household-level indicators of location (whether rural), number of household members, age, sex (whether female), and schooling years completed of the household head, and indicators of caste groups (scheduled caste, scheduled tribe, and other backward classes, as designated by the Indian government) and religion (Muslim, Christina, or Sikh). Finally, we measured the wealth of the household using a composite index (constructed by NFHS-5) of household assets such as TV, radio, and car, along with living condition indicators such as type of housing construction material and source of drinking water. We divided the wealth index into quintiles and included binary indicators of the top four wealth quintiles as covariates in the regression model, using the first (poorest) wealth quintile as the reference group.

The predicted probability from the regression model, known as propensity score, was then used to match adolescents in the post-COVID sample with similar adolescents in the pre-COVID sample. We used a one-to-one nearest neighbor matching algorithm (with replacement) which paired each post-COVID individual with a pre-COVID individual who had the closest propensity score value. We restricted our analysis to “common support”, i.e., individuals with overlapping propensity scores from the two groups. After matching, assuming that the unobserved characteristics of the post-COVID and matched pre-COVID samples were not systematically correlated with the outcome variable, the average difference in reading ability between the matched groups could be attributed to the pandemic. The difference is known as the “average treatment effect on the treated” or ATT^16–19,59^.

We estimated the ATT effect of the pandemic on reading ability and other outcomes discussed earlier for all adolescents and separately for boys and girls. For reading ability, we also conducted analysis separately for those from rural and urban areas, by caste and religious groups, and by wealth quintiles. Subgroup analyses for other outcome indicators are not reported due to low sample sizes. We report estimates which were statistically significant at 5%.

### Sensitivity analysis and matching quality tests

We tested the sensitivity of our results by using two additional analytical methods. The first was an alternative matching algorithm for PSM. Instead of matching a post-COVID individual with the nearest neighbor, we matched with three nearest neighbors in the post-COVID and calculated the ATT estimator from the matched sample. Second, we conducted an IPW regression analysis^60–62^. IPW is a commonly used regression technique that uses weights to balance the covariates between the intervention and control groups for producing statistically efficient standard errors. We used the propensity score estimated from the first stage of the PSM analysis and assigned a weight of the inverse of the propensity score for the intervention group. For the control group, the inverse of one minus the propensity score was assigned as the weight. We used a weighted linear probability model to regress the outcome variables on the set of covariates discussed earlier, along with a binary indicator of post-COVID status.

We tested if PSM successfully reduced the differences in the characteristics of the pre-COVID and post-COVID groups. We calculated the difference of the sample means between the two groups for each covariate used in PSM and divided it with the square root of the group difference variance^63^. This metric—known as the standardized percentage bias—should reduce substantially from pre- to post-matching.

### Reporting summary

Further information on research design is available in the Nature Research Reporting Summary linked to this article.

### Supplementary information

Supplementary Table 1

Reporting summary

## Data Availability

Data are publicly available from the Demographic and Health Surveys (DHS) Program https://dhsprogram.com/.

## Code availability

Code is available from the authors on request.
