# Supplementary material for: The gendered effects of the COVID-19 pandemic on adolescent literacy and schooling outcomes in India
Source: NPJ Sci Learn. 2023 Sep 22;8:42. doi: 10.1038/s41539-023-00193-8 (PMC10516864; doi:10.1038/s41539-023-00193-8)
Supplement: Supplementary file 1 — Supplementary Table 1 [file 41539_2023_193_MOESM1_ESM.docx]

Supplementary Table 1: Summary statistics of 15-17 year old samples in NFHS-5 and NFHS-4 data

|  | NFHS-5 (2019-2021) | | | NFHS-4 (2015-2016) | | | Difference in gender gap between NFHS-5 and NFHS-4 |
| --- | --- | --- | --- | --- | --- | --- | --- |
|  | 15-17 year old boys | 15-17 year old girls | Gender gap  (Boys – Girls) | 15-17 year old boys | 15-17 year old girls | Gender gap  (Boys – Girls) |  |
| Age in years | 15.97 | 15.985 | -0.010 | 15.990 | 15.974 | 0.016 | -0.026 |
| Years of schooling completed | 8.78 | 8.813 | -0.034 | 8.817 | 8.524 | 0.293 | -0.327 |
| *Relationship to household head:* |  |  |  |  |  |  |  |
| Self | 0.00 | 0.001 | 0.001 | 0.002 | 0.001 | 0.001 | -0.001 |
| Spouse | 0.00 | 0.004 | -0.004 | 0.000 | 0.006 | -0.006 | 0.002 |
| Daughter | 0.82 | 0.803 | 0.019 | 0.836 | 0.799 | 0.037 | -0.018 |
| Daughter-in-law | 0.00 | 0.021 | -0.020 | 0.001 | 0.028 | -0.028 | 0.007 |
| Grandchild | 0.13 | 0.123 | 0.012 | 0.109 | 0.105 | 0.004 | 0.008 |
| Age of household head | 48.59 | 48.520 | 0.068 | 48.487 | 48.420 | 0.067 | 0.001 |
| Whether household head is female | 0.17 | 0.159 | 0.012 | 0.144 | 0.136 | 0.008 | 0.004 |
| Household head’s years of schooling | 5.71 | 5.663 | 0.050 | 5.825 | 5.534 | 0.291 | -0.242 |
| *Household characteristics:* |  |  |  |  |  |  |  |
| Household size | 5.39 | 5.827 | -0.439 | 5.680 | 6.137 | -0.457 | 0.018 |
| Rural | 0.70 | 0.722 | -0.024 | 0.666 | 0.700 | -0.034 | 0.010 |
| Scheduled Caste (SC) | 0.22 | 0.234 | -0.013 | 0.210 | 0.217 | -0.007 | -0.006 |
| Scheduled Tribe (ST) | 0.10 | 0.098 | 0.003 | 0.090 | 0.095 | -0.005 | 0.008 |
| Other Backward Classes (OBC) | 0.44 | 0.428 | 0.007 | 0.444 | 0.439 | 0.004 | 0.003 |
| Muslim | 0.16 | 0.163 | -0.007 | 0.151 | 0.165 | -0.014 | 0.007 |
| Christian | 0.02 | 0.021 | -0.001 | 0.020 | 0.020 | 0.000 | 0.000 |
| Sikh | 0.02 | 0.013 | 0.003 | 0.014 | 0.012 | 0.001 | 0.002 |
| Wealth quintile 1 (poorest) | 0.22 | 0.237 | -0.020 | 0.162 | 0.201 | -0.039 | 0.020 |
| Wealth quintile 2 | 0.23 | 0.229 | 0.001 | 0.206 | 0.209 | -0.003 | 0.004 |
| Wealth quintile 3 | 0.20 | 0.204 | -0.002 | 0.201 | 0.207 | -0.005 | 0.003 |
| Wealth quintile 4 | 0.19 | 0.182 | 0.006 | 0.224 | 0.203 | 0.021 | -0.015 |
| Wealth quintile 5 (richest) | 0.16 | 0.149 | 0.015 | 0.207 | 0.180 | 0.027 | -0.012 |

Note: Data are from National Family Health Survey of India, 2019-2021 (NFHS-5) and 2015-2016 (NFHS-4). Mean values are presented.
